# The Impact of Serum Zinc Levels on Abdominal Fat Mass in Hemodialysis Patients

**DOI:** 10.3390/nu12030656

**Published:** 2020-02-28

**Authors:** Hirotaka Fukasawa, Hiroki Niwa, Kento Ishibuchi, Mai Kaneko, Takamasa Iwakura, Hideo Yasuda, Ryuichi Furuya

**Affiliations:** 1 Renal Division, Department of Internal Medicine, Iwata City Hospital, Iwata, Shizuoka 438-8550, Japan; 2 First Department of Medicine, Hamamatsu University School of Medicine, Hamamatsu, Shizuoka 438-8550, Japan

**Keywords:** hemodialysis, subcutaneous fat, visceral fat, zinc

## Abstract

Background: Zinc deficiency is highly prevalent and is caused by inadequate dietary intake, malabsorption and removal by treatment in hemodialysis patients. This study investigated the relationship between serum zinc levels and nutritional status in hemodialysis patients. Methods: A cross-sectional study examining 87 hemodialysis patients was performed. The serum concentrations of zinc were studied to evaluate their association with nutritional status, which was assessed by measuring abdominal muscle and fat areas with computed tomography. Results: Serum zinc levels were significantly and positively correlated with subcutaneous and visceral fat areas (*r* = 0.299, *p* < 0.01, and *r* = 0.298, *p* < 0.01, respectively), but not abdominal muscle areas. Multiple regression analyses demonstrated that serum zinc levels were a significant independent predictor of visceral fat areas (*p* < 0.01), but not subcutaneous fat areas (*p* = 0.631). Conclusions: Our findings suggest that serum zinc levels could play a crucial role in determining abdominal fat mass in hemodialysis patients.

## 1. Introduction

Zinc is an essential trace element for human nutrition, and its deficiency is associated with growth retardation, anorexia, insulin resistance, and impaired immune systems [1,2]. Patients undergoing hemodialysis (HD) have been reported to have low serum concentrations of zinc due to inadequate dietary intake, reduced gastrointestinal absorption, and zinc removal during HD sessions [3,4]. Serum zinc concentrations can also be reduced by increased expression of zinc transporter proteins by pro-inflammatory cytokines [5].

Most of HD patients suffer from nutritional problems that are associated with increased morbidity and mortality [6,7]. In fact, the body mass index (BMI) of HD patients is lower than that of age- and sex-matched control subjects [8]. Although overweight and obesity is shown to be significant risk factors for cardiovascular and all-cause mortality in the general population [9,10], a higher BMI is inversely associated with decreased mortality and a reduced risk of hospitalization in HD patients [11,12].

In this study, we showed the cross-sectional data from a well-characterized cohort of patients undergoing maintenance HD. In addition, we investigated the relationships between serum zinc levels and nutritional status by measuring abdominal muscle and fat areas in those patients.

## 2. Materials and Methods

### 2.1. Subjects

Eighty-seven patients (59 men, 28 women) who had been undergoing HD for at least three consecutive months at Iwata City Hospital (Shizuoka, Japan) were enrolled in this cross-sectional study. The causes of end-stage kidney disease were primary kidney diseases, such as chronic glomerulonephritis and nephrosclerosis in 60 patients (69%), polycystic kidney disease in six patients (7%), and overt diabetic nephropathy in 21 patients (24%). All patients had been subjected to regular HD for 4-4.5 h three times per week at a blood flow rate of 180-240 mL/min. All patients used bicarbonate dialysate (Kindaly AF-4E ^®^, Fuso, Osaka, Japan) at a dialysate flow rate of 500 mL/min. Patient’s information including the beginning date of HD, height, body weight, and comorbidities were collected from their medical records.

This study was approved by the institutional ethics committee and conducted in accordance with the Declaration of Helsinki. All of the participants provided written informed consent. This study was registered with the Clinical Trial Registry of the University Hospital Medical Information Network (http://www.umin.ac.jp/, study number: UMIN000039034).

### 2.2. Anthropometric Measurements

Body weight was measured before and after each dialysis session, and the post-dialysis body weight of each patient was used as his or her dry weight (DW). Height was also measured as each patient’s periodic evaluation. BMI (kg/m^2^) was calculated by dividing the DW (kg) by the squared height (m).

### 2.3. Blood Sampling and Laboratory Examinations

Blood samples were drawn at the beginning and end of the first dialysis session of the week, following a 2-day interval. Complete blood count was measured using XN-3000^TM^ hematology analyzer (Sysmex Corporation, Hyogo, Japan) and serum electrolytes, urea nitrogen, creatinine (Cr), albumin, cholesterol, and C-reactive protein (CRP) levels were measured using Ci16200 auto-analyzer (Canon Medical Systems, Tochigi, Japan). Serum zinc levels were measured using colorimetric methods (Espa Zn II kit; NIPRO, Osaka, Japan). A single-pool urea kinetic model was used to calculate the protein catabolic rate and the delivered dialysis dose, (clearance of urea (*K*; mL/min) multiplied by the time on dialysis (*t*; min) divided by the volume of distribution for urea (*V*_urea_; mL)), as described by Depner and Daugirdas [13].

### 2.4. Measurements of Abdominal Muscle and Fat Areas Using Computed Tomography

Computed tomography (CT) scans of the abdomen were performed during each patient’s periodic check-up. Each patient was imaged in the supine position and the thickness of each slice was 1.0mm. Axial CT images were used for muscle and fat mass evaluations at the level of the third lumber spine [14]. The abdominal muscle area (AMA), abdominal subcutaneous fat area (ASFA), and abdominal visceral fat area (AVFA) were automatically measured using SYNAPSE VINCENT software (version 3.0, Fujifilm Medical, Tokyo, Japan), which enables the tissue segmentation using Hounsfield unit thresholds [15]. To avoid the potentially confounding influence of body size, we also adjusted AMA, ASFA, and AVFA by dividing by the height of each patient (AMA/height, ASFA/height, and AVFA/height, respectively) [16].

### 2.5. Scoring System by Charlson Comorbidity Index

The Charlson comorbidity index was used to evaluate the status of comorbidities [17]. The index assigns 1 point for history of myocardial infarction, congestive heart failure, peripheral vascular disease, cerebrovascular disease, dementia, chronic pulmonary disease, connective tissue disorder, peptic ulcer disease, mild liver disease, and diabetes without end-organ damage; 2 points are assigned for hemiplegia, moderate to severe renal disease, diabetes with end-organ damage, tumor without metastases, leukemia, lymphoma, and myeloma; 3 points are assigned for moderate or severe liver disease; and 6 points are assigned for metastatic solid tumor or acquired immunodeficiency syndrome. For every decade >40 years of age, 1 point is added to the score. Because all patients were on dialysis, the minimum score is 2.

### 2.6. Statistical Analysis

Data were expressed as the mean ± standard deviation (SD) for continuous variables with normal distributions or the median and interquartile range (25th to 75th percentiles) for data with skewed distributions. The threshold for statistical significance was set at *p* < 0.05. Comparisons between two groups were performed using the Mann-Whitney *U*-test. Spearman’s rank-order correlation analyses were used to evaluate the potential associations between AMA/height, ASFA/height, and AVFA/height with the selected parameters including serum zinc levels. Multivariate regression analyses were used to assess the independent predictors of ASFA/height and AVFA/height. Independent variables were selected based on the results of correlation analyses (*p* < 0.05) in addition to age, gender and Charlson comorbidity index. CRP was also selected as a marker of inflammation. All statistical analyses were performed using IBM SPSS statistical software, version 25.0 (IBM SPSS, Tokyo, Japan).

## 3. Results

### 3.1. Clinical Profiles

Table 1 presents the characteristics of the study population. The median age was 68.0 years (the 25th to 75th percentile ranged from 60.0 to 71.5 years). The median dialysis vintage was 69.0 months (range, 37.0 to 221.5 months), and the mean BMI was 20.4 ± 3.6 kg/m^2^.

No significant sex differences were observed with respect to the age, dialysis vintage, BMI, Charlson comorbidity index, serum albumin, calcium, phosphate, zinc, and copper levels. The patient height, dry weight, and serum Cr levels were significantly greater in men than in women, whereas total cholesterol and LDL cholesterol levels were significantly greater in women than in men. In addition, AMA/height and AVFA/height were significantly greater in men than in women, whereas no significant sex differences were observed with respect to ASFA/height.

### 3.2. Correlations Between AMA/height, ASFA/height, and AVFA/height and Clinical Parameters

Significant positive correlations were observed between AMA/height and patient’s height (*p* < 0.001), dry weight (*p* < 0.001), BMI (*p* < 0.001), serum Cr levels (*p* < 0.001), ASFA/height (*p* < 0.05) and AVFA/height (*p* < 0.01). AMA/height was also negatively correlated with Kt/V_urea_ (*p* < 0.001, Table 2).

**Figure 1:**
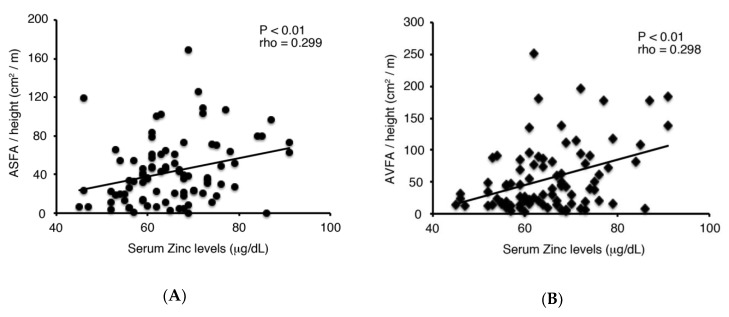
Correlations between serum Zinc levels and (**A**) ASFA standardized for height (ASFA/height, A) or (**B**) AVFA standardized for height (AVFA/height, B). Abbreviations: ASFA, abdominal subcutaneous fat area; AVFA, abdominal visceral fat area.

ASFA/height and AVFA/height were positively correlated with dry weight (*p* < 0.001), BMI (*p* < 0.001) and serum zinc levels (*p* < 0.01, Figure 1). Particularly, the strongest correlations were observed between BMI and ASFA/height or AVFA/height. ASFA/height and AVFA/height were also negatively correlated with dialysis vintage (*p* < 0.01 and *p* < 0.05, respectively). In addition, ASFA/height was positively correlated with serum albumin and LDL cholesterol levels (*p* < 0.05 and *p* < 0.01, respectively). On the other hand, significant positive correlations were observed between AVFA/height and serum Cr (*p* < 0.05), phosphate (*p* < 0.01) and β2-microglobulin levels (*p* < 0.05, Table 2).

### 3.3. Determinants of Abdominal Fat Areas

Multiple regression analyses revealed that serum zinc levels were significantly associated with both ASFA/height and AVFA/height, when serum zinc levels, age, gender, and dialysis vintage were included as independent variables (Model 1, Table 3). Furthermore, serum zinc levels were significantly associated with AVFA/height, when Charlson comorbidity index, BMI, LDL cholesterol, and CRP were added as independent variables (Model 2).

## 4. Discussion

The primary finding of this study is that serum zinc levels are significantly associated with abdominal fat mass in HD patients. To our knowledge, this is the first report to show the relationship between serum zinc levels and the nutritional status assessed by measuring abdominal muscle and fat areas in patients with advanced CKD.

Advanced CKD patients are often suffering from nutritional problems, which are associated with increased morbidity and mortality [6,7]. Actually, the body mass index (BMI) in hemodialysis (HD) patients exhibits lower than age- and sex-matched control subjects [8]. Previous studies showed that higher BMIs contributed to a survival advantage in HD patients [11,18]. Because higher BMIs are associated with an increased risk of cardiovascular disease (CVD) and mortality in the general population [9], this reverse relationship observed in HD patients is known as the “risk factor paradox” or “reverse epidemiology” [19,20]. Besides, it is unclear whether this survival advantage associated with higher BMIs in HD patients is caused by increased muscle mass, fat mass, or both. One reason why this question remains unclear is because BMIs do not distinguish between muscle mass and adipose tissue [11]. Previously, Beddhu et al. [21] attempted to solve this problem using 24-h urinary creatinine excretion as a marker for muscle mass in conjunction with BMIs and proposed that muscle mass might be more important in this survival advantage than adipose tissue. On the other hand, Caetano et al. [22] reported that adipose tissue might be more important than muscle mass in predicting 1-year mortality using bioimpedance spectroscopy.

In the present study, we showed that serum zinc levels were positively and significantly correlated with the abdominal fat areas in HD patients. In the previous study, it is reported that zinc stimulates the differentiation of pre-adipocytes to adipocytes in vitro [23]. Another report showed that chronic zinc supplementation induced the increased size of adipocytes resulted in the adipose tissue hypertrophy in mice [24]. Zhang et al. [25] reported that dietary zinc supplementation increased intramuscular adipose deposition in piglets. Chen et al. [26] also reported that zinc supplementation for 6 weeks induced body fat accumulation in genetically obese mice and dietary-obese mice. These previous reports support our results, although further studies are needed to clarify the mechanism how zinc in the serum affects the fat accumulation and the metabolism of adipose tissue in HD patients.

One of the key findings in this study is that serum zinc levels may be a predictor of visceral fat areas in the abdomen of HD patients, even after adjusting for potential confounding variables, but not subcutaneous fat areas (Model 2 in Table 3). Previously, Huang et al. [24] reported that long-term chronic zinc supplementation induced the accumulation of visceral adipose tissue, but not subcutaneous adipose tissue in mice, which supports our results. A large-scale study showed that the absolute value of visceral fat correlated with obesity-related cardiovascular risks, although cardiovascular risks did not increase with the increase of subcutaneous fat [27]. Adipose tissues in subcutaneous fat obesity might function normally with the expected release of anti-inflammatory adipokines, whereas adipose tissues in visceral fat obesity release an increased amount of pro-inflammatory adipokines and suppress the secretion of anti-inflammatory adipokines, thereby creating low-grade inflammation, which contributes to systemic metabolic and cardiovascular diseases [10,28]. Accordingly, each character of visceral and subcutaneous adipose tissues is quite different in the obesity. On the other hand, it is reported that BMI in HD patients is much lower than age- and sex-matched control subjects [8]. In addition, Yajima et al. [29] reported that higher visceral fat areas and lower subcutaneous fat areas tended to be associated with a reduced risk for all-cause mortality in HD patients. Taken together, it remains unclear whether the difference of abdominal fat distribution plays a role in the risk for mortality and how zinc in the serum affects abdominal fat distribution in HD patients. Further studies are needed to answer these enigmas.

Our study has several limitations. First, due to the cross-sectional study design, a longitudinal causal relationship cannot be established between the changes in serum zinc levels and alterations in abdominal fat levels. Second, because of the relatively small number of patients in our cohort, the generalizability of our conclusions remains unclear. Third, serum zinc concentrations are affected by several factors including meals and the blood sampling time, although the fluctuation range is less than 10% [30]. To minimize those effects, we collected blood samples under similar conditions, just before the hemodialysis treatment.

## 5. Conclusions

In conclusion, serum zinc levels are significantly and positively correlated with abdominal fat areas in HD patients. Our findings suggest that zinc can play an important role in determining the nutritional status of HD patients, although our data should be confirmed by larger studies. In addition, future longitudinal observations and interventional studies are warranted to establish whether this link is causal in nature.

## Figures and Tables

**Table 1:** Patient characteristics.

	Total (*n* = 87)	Men (*n* = 59)	Women (*n* = 28)
Age, years	68.0 (60.0 to 71.5)	69.0 (62.0 to 73.0)	62.0 (56.5 to 70.0)
Dialysis vintage, months	69.0 (37.0 to 221.5)	64.0 (35.0 to 212.0)	106.5 (46.5 to 259.8)
Height, m	1.61 ± 0.09	1.65 ± 0.07	1.52 ± 0.06 ^c^
Dry weight, kg	53.0 ± 11.5	56.1 ± 11.5	46.3 ± 8.3 ^c^
BMI, kg/m^2^	20.4 ± 3.6	20.6 ± 3.6	19.9 ± 3.5
Charlson comobidity index	4.1 ± 1.7	4.3 ± 1.7	3.8 ± 1.8
Hemoglobin, g/dL	11.5 ± 1.7	11.6 ± 1.2	11.3 ± 0.9
Total protein, g/dL	7.4 ± 0.7	7.4 ± 0.6	7.4 ± 0.7
Serum albumin, g/dL	4.2 ± 0.4	4.2 ± 0.4	4.1 ± 0.5
Total cholesterol, mg/dL	146.5 ± 32.5	137.1 ± 29.2	166.2 ± 30.6 ^c^
LDL choresterol, mg/dL	74.3 ± 22.6	68.9 ± 21.1	85.7 ± 21.8 ^b^
Blood urea nitrogen, mg/dL	60.8 ± 15.0	61.9 ± 15.1	58.7 ± 14.7
Serum creatinine, mg/dL	10.9 ± 2.6	11.5 ± 2.6	9.5 ± 2.1 ^c^
Calcium, mg/dL	8.9 ± 0.4	8.8 ± 0.4	9.0 ± 0.4
Phosphate, mg/dL	5.6 ± 1.2	5.6 ± 1.1	5.8 ± 1.4
Intact PTH, pg/mL	109.9 ± 97.9	107.6 ± 101.6	114.9 ± 91.2
b_2_-microglobulin, mg/L	27.6 ± 5.3	27.8 ± 5.4	27.2 ± 5.0
Kt/V _urea_	1.6 ± 0.3	1.5 ± 0.2	1.8 ± 0.3 ^c^
nPCR, g/kg/ideal body weight/day	0.93 ± 0.20	0.93 ± 0.20	0.93 ± 0.20
CRP, mg/dL	0.00 (0.00 to 0.24)	0.13 (0.00 to 0.37)	0.00 (0.00 to 0.11) ^b^
Zinc, mg/dL	64.0 (59.0 to 71.5)	64.0 (59.0 to 71.0)	65.0 (59.0 to 71.3)
AMA, cm^2^	80.3 (68.9 to 100.2)	90.8 (76.0 to 107.7)	68.8 (62.3 to 74.8) ^c^
AMA standardized for height	51.2 (44.0 to 59.8)	54.7 (47.2 to 65.3)	44.9 (40.4 to 49.6) ^c^
ASFA, cm^2^	57.7 (29.6 to 99.6)	51.9 (22.2 to 99.3)	64.0 (32.9 to 98.8)
ASFA standardized for height	36.6 (18.4 to 61.1)	33.4 (13.5 to 60.9)	41.0 (23.0 to 63.4)
AVFA, cm^2^	59.2 (25.5 to 134.6)	81.9 (27.7 to 147.6)	33.1 (24.5 to 47.6) ^b^
AVFA standardized for height	38.5 (16.4 to 82.3)	48.9 (17.6 to 86.8)	21.5 (15.3 to 32.5) ^b^

^b, c^ Significantly different from men, *p* < 0.01 and *p* < 0.001, respectively. All variables were expressed as the mean ± SD or the median and interquartile range (25th to 75th percentiles). Abbreviations: AMA, abdominal muscle area; ASFA, abdominal subcutaneous fat area; AVFA, abdominal visceral fat area; BMI, body mass index; CRP, C-reactive protein; Kt/V _urea_, amount of dialysis delivered to each patient per treatment; LDL, low-density lipoprotein; nPCR, normalized protein catabolic rate; PTH, parathyroid hormone.

**Table 2:** Correlations between AMA standardized for height (AMA/height), ASFA standardized for height (ASFA/height), AVFA standardized for height (AVFA/height) and clinical parameters.

	AMA/Height		ASFA/Height		AVFA/Height	
	Correlation Coefficient	*p*	Correlation Coefficient	*p*	Correlation Coefficient	*p*
Age	−0.156	0.149	−0.064	0.559	0.200	0.064
Dialysis vintage	−0.191	0.077	−0.361	<0.01	−0.248	<0.05
Height	0.458	<0.001	−0.039	0.720	0.183	0.089
Dry weight	0.640	<0.001	0.558	<0.001	0.692	<0.001
BMI	0.531	<0.001	0.725	<0.001	0.752	<0.001
Charlson comorbidity index	0.045	0.676	0.069	0.525	0.113	0.298
Hemoglobin	0.008	0.943	0.105	0.334	0.204	0.058
Total protein	0.155	0.153	0.210	0.051	0.194	0.072
Serum albumin	0.087	0.422	0.232	<0.05	0.103	0.342
Total cholesterol	−0.138	0.232	0.041	0.725	−0.130	0.259
LDL cholesterol	0.020	0.854	0.331	<0.01	0.178	0.105
Blood urea nitrogen	0.119	0.273	−0.054	0.620	0.025	0.820
Serum creatinine	0.440	<0.001	0.145	0.181	0.226	<0.05
Calcium	−0.114	0.292	0.091	0.402	−0.032	0.770
Phosphate	0.129	0.234	0.090	0.406	0.345	<0.01
Intact PTH	0.073	0.501	0.043	0.695	0.061	0.597
_2_-microglobulin	−0.007	0.951	−0.073	0.507	0.232	<0.05
Kt/V _urea_	−0.624	<0.001	−0.145	0.182	−0.351	<0.01
nPCR	0.011	0.920	−0.035	0.746	0.014	0.900
CRP	0.039	0.72	−0.077	0.478	0.134	0.216
Zinc	0.196	0.069	0.299	<0.01	0.298	<0.01
AMA	0.978	<0.001	0.178	0.100	0.330	<0.01
AMA/height	-	-	0.226	<0.05	0.348	<0.01
ASFA	0.259	<0.05	0.998	<0.001	0.757	<0.001
ASFA/height	0.226	<0.05	-	-	0.746	<0.001
AVFA	0.357	<0.01	0.739	<0.001	0.998	<0.001
AVFA/height	0.348	<0.01	0.746	<0.001	-	-

The numbers of coefficient represent the correlation coefficients. Abbreviations: AMA, abdominal muscle area; ASFA, abdominal subcutaneous fat area; AVFA, abdominal visceral fat area; BMI, body mass index; CRP, C-reactive protein; Kt/V _urea_, amount of dialysis delivered to each patient per treatment; LDL, low-density lipoprotein; nPCR, normalized protein catabolic rate; PTH, parathyroid hormone.

**Table 3:** Multiple regression analysis with ASFA standardized for height or AVFA standardized for height as the dependent variables and serum Zinc levels, age, gender, dialysis vintage, BMI, LDL cholesterol, and CRP as the independent variables.

	Model 1		Model 2	
	*β*	*p*	*β*	*p*
Dependent variable: ASFA standardized for height				
Zinc, mg/dL	0.263	<0.01	0.031	0.631
Age, years	0.065	0.520	0.080	0.202
Gender, male	−0.250	<0.05	−0.245	<0.001
Dialysis vintage, months	−0.346	<0.01	−0.171	<0.01
Charlson comorbidity index	-	-	−0.034	0.597
BMI, kg/m^2^	-	-	0.766	<0.001
LDL choresterol, mg/dL	-	-	0.073	0.277
CRP, mg/dL	-	-	−0.005	0.944
Dependent variable: AVFA standardized for height				
Zinc, mg/dL	0.375	<0.001	0.198	<0.01
Age, years	0.094	0.352	0.115	0.085
Gender, male	0.204	<0.05	0.164	<0.05
Dialysis vintage, months	−0.173	0.083	−0.033	0.629
Charlson comorbidity index	-	-	−0.058	0.397
BMI, kg/m^2^	-	-	0.675	<0.001
LDL choresterol, mg/dL	-	-	0.075	0.294
CRP, mg/dL	-	-	−0.058	<0.05

Abbreviations: ASFA, abdominal subcutaneous fat area; AVFA, abdominal visceral fat area; BMI, body mass index; CRP, C-reactive protein; LDL, low-density lipoprotein.
